# Signal Recovery from Randomly Quantized Data Using Neural Network Approach

**DOI:** 10.3390/s22228712

**Published:** 2022-11-11

**Authors:** Ali Al-Shaikhi

**Affiliations:** 1 Department of Electrical Engineering, King Fahd University of Petroleum & Minerals, Dhahran 31261, Saudi Arabia; shaikhi@kfupm.edu.sa; 2 Center for Communication Systems and Sensing, King Fahd University of Petroleum & Minerals, Dhahran 31261, Saudi Arabia

**Keywords:** quantization, deconvolution, LSTM neural network

## Abstract

We present an efficient scheme based on a long short-term memory (LSTM) autoencoder for accurate seismic deconvolution in a multichannel setup. The technique is beneficial for compressing massive amounts of seismic data. The proposed robust estimation ensures the recovery of sparse reflectivity from acquired seismic data that have been under-quantized. By adjusting the quantization error, the technique considerably improves the robustness of data to the quantization error, thereby boosting the visual saliency of seismic data compared to the other existing algorithms. This framework has been validated using both field and synthetic seismic data sets, and the assessment is carried out by comparing it to the steepest decent and basis pursuit methods. The findings indicate that the proposed scheme outperforms the other algorithms significantly in the following ways: first, in the proposed estimation, fraudulently or overbearingly estimated impulses are significantly suppressed, and second, the proposed guesstimate is much more robust to the quantization interval changes. The tests on real and synthetic data sets reveal that the proposed LSTM autoencoder-based method yields the best results in terms of both quality and computational complexity when compared with existing methods. Finally, the relative reconstruction error (RRE), signal-to-reconstruction error ratio (SRER), and power spectral density (PSD) are used to evaluate the performance of the proposed algorithm.

## 1. Introduction

Seismic deconvolution is the process of removing a wavelet from the recorded seismic traces, which also diminishes short-period multiples and repercussions. Seismic deconvolution is an important component of the seismic data processing pipeline because it produces a more interpretable seismic section. Deconvolution techniques are widely adopted in seismic applications and beyond [1]. Blind deconvolution is a common challenge in seismic deconvolution because the blurring kernel, which is the source seismic wavelet, is unidentified [2].

In a seismic survey, the wave field originated from a seismic source and received by an array of sensors ultimately produces a seismic data section. Usually, in exploration geophysics, we deal with a multichannel scenario [3,4,5], in which seismic traces are commonly represented as convolutions of the same source waveform with multiple channels stemming from various reflectivity models. In the multichannel blind deconvolution application, conventional approaches do not produce satisfactory results for a number of reasons. The primary reason is the high similarity among neighboring reflectivity series, which either makes the issue numerically sensitive or, in the worst-case scenario, hard to solve and ill-posed [6].

In order to address this problem, ref. [7] proposed a Bayesian approach in which a regularization term based on a hybrid $\ell_{1}/\ell_{2}$-norm is adopted for sparsity promotion. This method, called the steepest decent (SD) approach, performs admirably with both field and synthetic data sets. However, its high computational complexity prevents it from being adapted to industrial-scale data sets. The other solution is a series of deconvolution filtering concept variations, such as the commonly used deconvolution prediction filter [8] and the fast sparse method for blind multichannel deconvolution [6]. These techniques yield a deconvolution filter based on an established criterion—for example, the $\ell_{1}/\ell_{2}$-norm or the least-squares of the deconvoluted signal. Their computational cost is typically far lower compared to the other methods. However, the limited filter length becomes a bottleneck in their performance. For instance, the Wiener filter will increase the noise in the process of deconvolution, and this method cannot produce a wide band spiky deconvolution result.

This work considers a realistic application in which the recorded seismic time-series traces are quantized element-by-element in an attempt to lessen storage and communication costs. The quantization is performed on the randomly selected samples in a trace. According to lossy compression methods, for example, [9,10,11,12], the higher the compression gain, the lower the reconstruction quality. Consequently, depending on the quantization interval, the accuracy of predicting the reflectivity series from quantized traces is jeopardized. To alleviate this annoyance, we propose a robust optimization scheme that improves prediction robustness and accuracy.

The proposed scheme is an efficient deconvolution method that utilizes long short-term memory (LSTM) autoencoder networks [13] by exploiting the multichannel structure of the seismic section and band-limited/sparse characteristics of the seismic source wavelet [14]. Furthermore, it is a type of recurrent neural network that can learn long-term dependencies in data. The seismic trace is well-suited for this kind of architecture due to the high correlation between the samples. In this work, the parameters (number of layers, weights, biases) in LSTM are selected based on the selectivity analysis. The LSTM itself has not been modified or improved. The quantized data are used to recover the seismic reflectivity. Simulations with both field and synthetic data sets illustrate that this novel approach gives fast and high-quality deconvolution results. In comparison with prevailing methods, we show examples where the proposed method outperforms the SD method and the basis pursuit (BP) method (discussed in Section 3) in both deconvolution results and computational efficiency.

*Notation*: Vectors/Matrices are represented by bold notations, whereas scalars are represented by lower-case letters. The rest of this paper is organized as follows: Section 2 and Section 3 are devoted to the convolution model and the traditional deconvolution approaches, respectively. The proposed deconvolution approach is presented in Section 4, whereas the experimental results and discussion are reported in Section 5. Finally, Section 6 draws the conclusions.

## 2. Convolution Model

**Figure 1:**
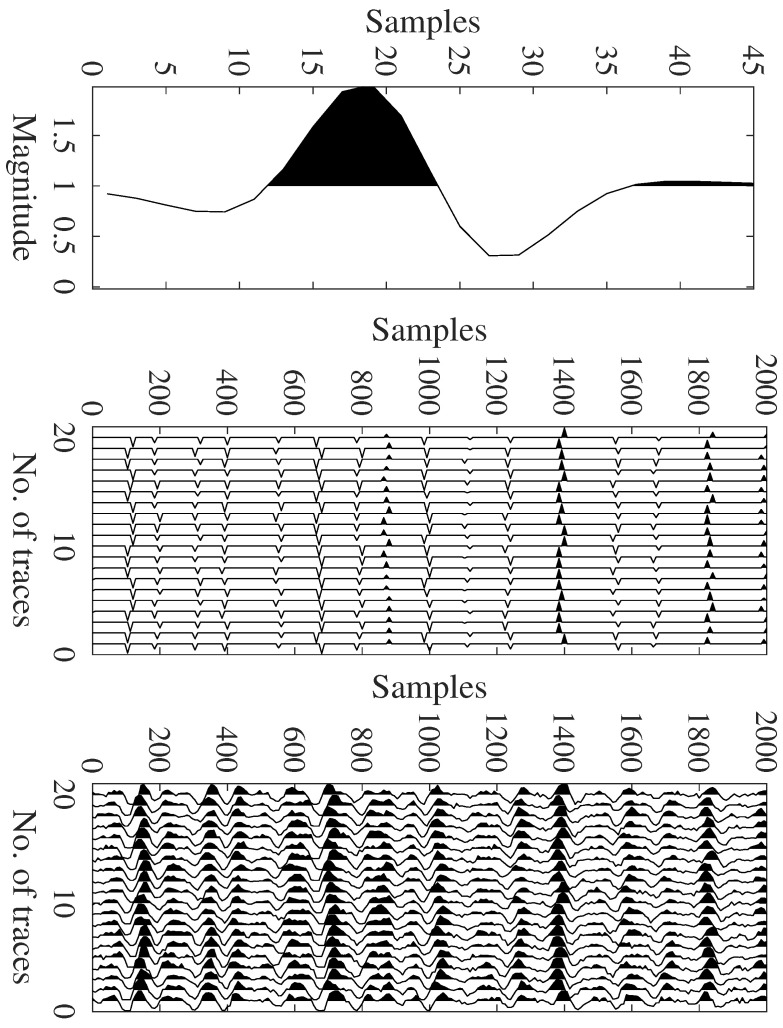
Top to bottom: a wavelet, the reflectivity series, and the recorded traces.

The recorded seismic trace can be modeled as a linear system [15] in which the Earth’s impulse response is convoluted with a seismic source wavelet. The *j*-th received seismic trace, in particular, is given as follows [16]:

(1) $$\begin{array}{cc} x_{j}\left[n\right] & =w\left[n\right]*r_{j}\left[k\right]+z_{j}\left[n\right]=\sum_{k}w\left[n-k\right]r_{j}\left[k\right]+z_{j}\left[n\right],\;j=1,\ldots ,J, \end{array}$$

where ∗ represents the convolution operation, w[n] denotes the source wavelet and $r_{j}\left[n\right]$ denotes the reflectivity model with *J* number of channels. Figure 1 gives the pictorial view of the convolutional model.

Before delving into the effect of noise $z_{j}\left[n\right]$, we concentrate on the noiseless case. The *z*-transform of $x_{j}\left[n\right]$ in the noiseless version of (1), i.e., $z_{j}\left[n\right]=0$, gives [7]:

(2) $$X_{j}\left(z\right)=W\left(z\right)R_{j}\left(z\right).$$

Similarly, the *z*-transform of $x_{i}\left[n\right]$ gives:

(3) $$X_{i}\left(z\right)=W\left(z\right)R_{i}\left(z\right).$$

Multiplying (2) and (3) by $R_{i}\left(z\right)$ and $R_{j}\left(z\right)$, respectively, we can conclude that the succeeding system of linear equations can be acquired by contemplating (2) for a couple of traces:

(4) $$X_{i}\left(z\right)R_{j}\left(z\right)-X_{j}\left(z\right)R_{i}\left(z\right)=0,\;\mathrm{for}\;i\neq j,$$

which can be rewritten in matrix form as follows:

(5) $$X_{i}r_{j}-X_{j}r_{i}=0,$$

whereas $X_{j}$ represents the *j*-th channel convolution matrix, $r_{j}={\left[r_{j}\left[1\right],\ldots ,r_{j}\left[N\right]\right]}^{T}$ is the vectorized reflectivity and *N* represents the samples in a trace. We can combine all instances of Equation (5) into the one equation as follows: [6]

(6) Ar=0,

where

(7) $$A=\left[\begin{array}{cccccc} X_{2} & -X_{1} & \; & & \; & \\ X_{3} & \; & -X_{1} & \; & & \\ X_{4} & \; & & -X_{1} & \; & \\ \vdots & \; & & \; & \ddots & \\ \; & X_{3} & -X_{2} & \; & & \\ \; & X_{4} & \; & -X_{2} & \; & \\ \; & X_{5} & \; & & -X_{2} & \\ \; & \vdots & \; & & \; & \ddots \end{array}\right]$$

and $r={\left[r_{1}^{T},r_{2}^{T},\ldots ,r_{J}^{T}\right]}^{T}$. The modification for the additive noise scenario is [6]:

(8) Ar=e,

but the noise vector e is neither white nor Gaussian [7].

The direct benefit of using less quantization levels is that it compresses the seismic data volume. In this case, the compression gain is defined as the number of bits used by the original seismic trace divided by the number of bits used by the quantized seismic trace. It is calculated as follows: if *N* binary bits are used to store each sample of the original seismic trace, then the quantization interval is defined as q=δY, where 0<δ≤1 is the scaling factor and *Y* is the sample’s maximum absolute value. Hence, the codewords are (2/δ)+1 in size, and each quantized seismic sample is represented by $\left\lceil \log_{2}\left[\left(2/\delta \right)+1\right]\right\rceil$ number of bits. Eventually, $C_{r}$, the compression gain is given by:

(9) $$\begin{array}{c} C_{r}\left(q,N\right)=\frac{N}{\log_{2}\left[\left(2Y/q\right)+1\right]}\approx \frac{N}{\log_{2}2Y}\log_{2}q \end{array}$$

Despite the fact that compression gain increases exponentially with the *q* value, as can be seen from (9), the quantization error, which is defined as the difference between the quantized and original samples, i.e., $e_{i}=x_{i}-x_{i}^{\prime }$, has an upper-bound determined by the *q* value:

(10) $$\begin{array}{c} \left|e_{i}\right|\leq \frac{1}{2}q \end{array}$$

and, similarly, the $l_{2}$-norm of the error quantization vector, $e=x-x^{\prime }$:

(11) $$\begin{array}{cc} \; & {∥e∥}_{2}\leq \frac{1}{2}q\sqrt{m} \end{array}$$

where *m* is the number of samples in a trace. The above Equation (11) pertains to a trade-off among the increased compression gain and adequate preservation of the signal quality, which applies to all lossy compression strategies. We link the two options of conundrum with that of the value of *q* under an application domain, namely, reflectivity estimation. This effectively calibrates the trade-off between accuracy and compression in this study. By integrating the quantization into the convolution model, the seismic quantized trace is characterized as a linear system with admixture bounded noise:

(12) $$\begin{array}{c} x^{\prime }=wr+e \end{array}$$

We are interested in locating a sparse linear combination of basic elementary signals which is impacted by the confined quantization error. To be precise, the work is understood as follows: given an observed signal $x^{\prime }$ and its linear model in (12), approximate the coefficient vector r.

## 3. Deconvolution Methods

**Figure 2:**
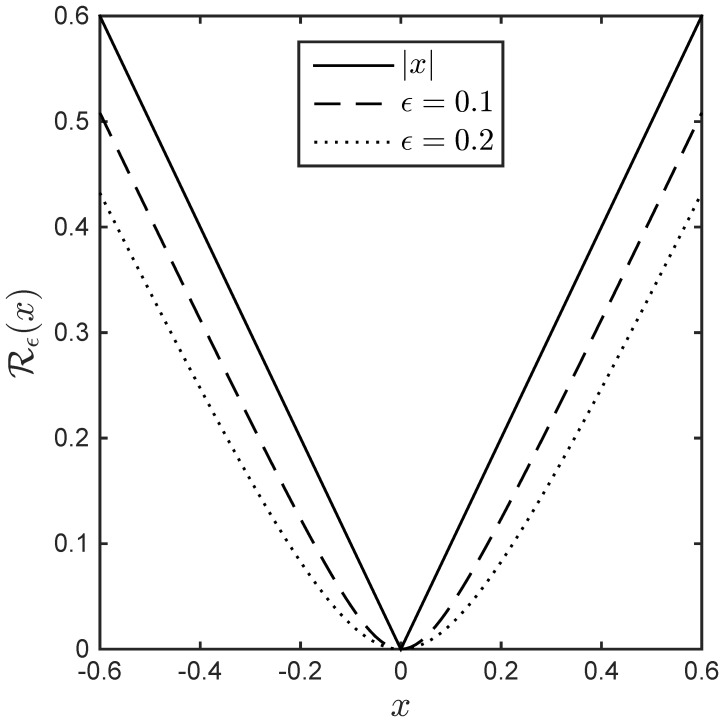
$\ell_{1}/\ell_{2}$-norm $R_{\epsilon }\left(x\right)$ for different ϵ values.

In this section, we look at some traditional techniques that will be used as benchmarks for our proposed approach. We divide these methods into two classes, i.e., SD and BP methods. Our aim in these methods is to optimize [17]:

(13) $$\hat{r}=\underset{r}{\mathrm{argmin}}\left\{\frac{1}{2}{∥Ar∥}_{2}^{2}+\lambda \sum_{j}R_{\epsilon }\left(r_{j}\right)\right\},\mathrm{subject}\;\mathrm{to}r^{T}r=1,$$

where the constraint $r^{T}r=1$ is added to avoid a trivial solution in (13). The regularization term is a differentiable hybrid $\ell_{1}/\ell_{2}$-norm, which makes the optimization easier, and $R_{\epsilon }\left(r_{j}\right)=\sum_{n}\left(\sqrt{r_{j}^{2}\left[n\right]+\epsilon_{k}^{2}}-\epsilon_{k}\right)$, which approximates an $\ell_{1}$-norm and is added in order to promote the sparsity of the output $\hat{r}$. As illustrated in Figure 2, small values of ϵ generate a mixed norm that can be very close to the $\ell_{1}$ norm.

In order to honor the constraint $r^{T}r=1$ in computation, [7] introduced a variant of iterative steepest descent which employs Rodrigues’s rotation formula [18]. The resulting updated equation for the SD method is given as [17]:

(14) $${\hat{r}}^{\left(i+1\right)}=\cos\left(\theta^{\left(i\right)}\right){\hat{r}}^{\left(i\right)}+\sin\left(\theta^{\left(i\right)}\right)\nabla L^{\left(i\right)}/\left|\nabla L^{\left(i\right)}\right|,$$

where

(15) $$\begin{array}{ccc} \nabla L^{\left(i\right)} & = & \underset{{}^{h}}{\underbrace{A^{T}A{\hat{r}}^{\left(i\right)}+\lambda \left\{{\hat{r}}^{T(i)}⊙{\hat{r}}^{\left(i\right)}+1\epsilon_{k}^{2}\right\}⊙{\hat{r}}^{\left(i\right)}}}\\ & & \;-{\hat{r}}^{(i)T}\left[A^{T}A{\hat{r}}^{\left(i\right)}+\lambda \left\{{\hat{r}}^{T(i)}⊙{\hat{r}}^{\left(i\right)}+1\epsilon_{k}^{2}\right\}⊙{\hat{r}}^{\left(i\right)}\right]{\hat{r}}^{\left(i\right)} \end{array}$$

and $\theta^{\left(i\right)}=-\alpha /(h^{T}\nabla L^{\left(i\right)}/|\nabla L^{\left(i\right)}\left|\right)$. The notation ⊙ represents the element-by-element multiplication of two vectors, and 1 is a vector of all ones with length equal to ${\hat{r}}^{\left(i\right)}$.

Another solution is to design a filter (v[n]) for deconvolution that minimizes the $\ell_{1}/\ell_{2}$-norm of the deconvolved trace ${\hat{r}}_{j}\left[n\right]=\sum_{k}v\left(k\right)x_{j}\left(n-k\right)=x_{j}\left[n\right]*v\left[n\right]$. Thus, we solve the following optimization problem [7]:

(16) $$\hat{v}=\underset{v}{\mathrm{argmin}}\sum_{j}R_{\epsilon }\left(r_{j}\right),\mathrm{subjet}\mathrm{to}v^{T}v=1,$$

where the sparse-promoting norm $R_{\epsilon }$ is defined as $R_{\epsilon }\left(y_{j}\right)=\sum_{n}\left(\sqrt{\frac{r_{j}^{2}\left(n\right)}{\sigma_{y_{j}}^{2}}+\epsilon_{f}^{2}}-\epsilon_{f}\right)$ and $\sigma_{y_{j}}^{2}=\sum_{n}r_{j}^{2}\left(n\right)/N$ is the predicted variance obtained from the estimated reflectivity series. The conditionality vTv=1 is levied to prevent the trivial solution.

The iterative SD algorithm provides an accurate solution to problem (16). The updated equation is:

(17) $$\begin{array}{ccc} {\hat{v}}^{\left(i+1\right)}= & {\hat{v}}^{\left(i\right)}-\mu \nabla L^{\left(i\right)},\;{\hat{v}}^{\left(i+1\right)}= & \frac{{\hat{v}}^{\left(i+1\right)}}{∥{\hat{v}}^{\left(i+1\right)}{∥}_{2}} \end{array}$$

where μ is the step size of the gradient algorithm and

(18) $$\begin{array}{cc} \nabla L_{k}^{\left(i\right)}= & \sum_{j}\sum_{n}\frac{{\left\{{\hat{r}}_{j}^{2(i)}\left(n\right)/\sigma_{{\hat{r}}_{j}^{\left(i\right)}}^{2}+\epsilon_{f}^{2}\right\}}^{-1/2}}{\sigma_{{\hat{r}}_{j}^{\left(i\right)}}^{4}}Q_{k}^{\left(i\right)}\left(j,n\right)\\ Q_{k}^{\left(i\right)}\left(j,n\right)= & \;\sigma_{{\hat{r}}_{j}^{\left(i\right)}}^{2}{\hat{r}}_{j}^{\left(i\right)}\left(n\right)x_{j}\left(n-k\right)-\frac{{\hat{r}}_{j}^{2(i)}\left(n\right)}{N}\sum_{n}{\hat{r}}_{j}^{\left(i\right)}\left(n\right)x_{j}\left(n-k\right). \end{array}$$

Compared with the first approach, the number of parameters in the second approach (i.e., the length of the deconvolution filter) is much less; as a result, the second approach’s computational complexity is substantially lower as compared to the first one.

Another method, called the basis pursuit method [17], is implemented in the following three steps:

1. Initial peak detection for each channel. For the *j*-th trace, we find an initial reflectivity $r_{j}^{0}$ via:
  (19) $$r_{j}^{0}=\underset{r_{j}}{\mathrm{argmin}}{\left({\bar{x}}_{j}^{T}r_{j}-1\right)}^{2}\;\mathrm{subject}\;\mathrm{to}{∥r_{j}∥}_{1}=\tau .$$
  where ${\bar{x}}_{j}$ is the *j*-th received trace normalized by its maximal absolute value.
2. Wavelet estimation.
  The assumption that the wavelet is identical for all the channels is now exploited. A seismic source is usually band-limited in reality, so the sparsity of the wavelet in the frequency domain is a reasonable assumption. The Fourier transform of the seismic trace, $x_{j}\left[n\right]$ in (1)
  (20) $$X_{j}\left(e^{j\omega }\right)=R_{j}\left(e^{j\omega }\right)W_{j}\left(e^{j\omega }\right)+Z_{j}\left(e^{j\omega }\right),$$
  would be sampled in the frequency domain with a DFT, represented by F{·}, in order to create length-$N_{\mathrm{DFT}}$ vectors $W_{j}$, $R_{j}$ and $X_{j}$, After replacing $R_{j}$ with the DFT of the initial estimate of reflectivity, $R_{j}^{0}=F\left\{r_{j}^{0}\right\}$, the following minimization problem can be solved for the estimated wavelet in each channel:
  (21) $${\tilde{W}}_{j}=\mathrm{argmin}∥W_{j}{∥}_{1}\;\mathrm{subject}\;\mathrm{to}\;∥X_{j}-\mathrm{diag}\left(R_{j}^{0}\right)W_{j}{∥}_{2}\leq {∥Z_{j}∥}_{2},$$
  where $\mathrm{diag}(R_{j}^{0})$ is a diagonal matrix with entries from the vector $R_{j}^{0}$.
  Once an estimate of the wavelet is obtained for each channel, stacking is used to improve the SNR of the common wavelet estimate [8]. The stacking process is carried out using ${\tilde{W}}_{j}$ over all channels to obtain a single estimated wavelet $\tilde{w}$ in the time domain:
  (22) $$\tilde{w}=ℜ\left[F^{-1}\left\{\frac{1}{J}\sum_{i=1}^{J}{\tilde{W}}_{j}\right\}\right],$$
  where *ℜ* represents the real part.
3. Refining the estimate of the reflectivity model. The following basis pursuit denoising problem is solved in order to estimate a sparse reflectivity series:
  (23) $${\hat{r}}_{j}=\mathrm{argmin}∥r_{j}{∥}_{1}\;\mathrm{subject}\mathrm{to}\;∥x_{j}-Cr_{j}{∥}_{2}\leq {∥z_{j}∥}_{2},$$
  where C represents the convolution matrix having entries related to $\tilde{w}$ and $x_{j}={\left[x_{j}\left[1\right],\ldots ,x_{j}\left[N\right]\right]}^{T}$.

## 4. Neural Network Architecture

**Figure 3:**
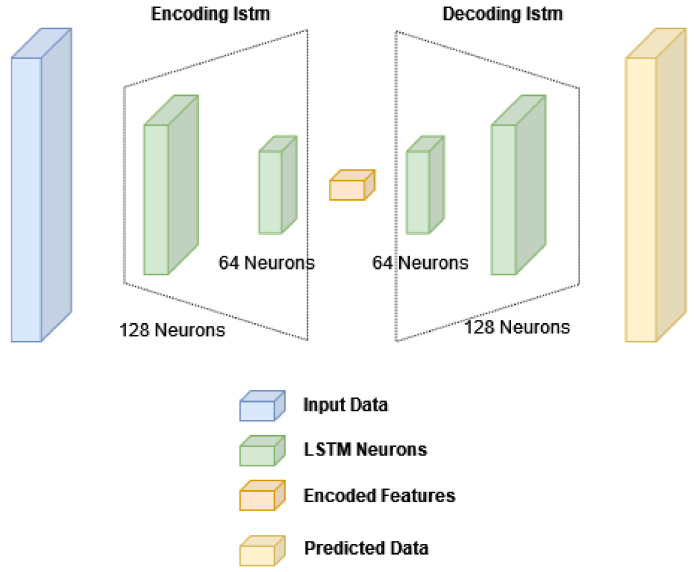
LSTM neural network for SNR enhancement with convolutional (Conv) and LeakyRelu layers.

The proposed method that makes use of the LSTM neural network-based autoencoder is to be explained in this section. The network architecture is shown in Figure 3. It consists of LSTM layers for performing the encoder/decoder functions.

The LSTM layer in time series and sequence data learns protracted correlations among time steps. The layer state comprises the cell state $c_{t}$ and the hidden state (also known as the output state) $h_{t}$. The output of the LSTM layer for time step *t* is stored in the hidden state at that time. Information from previous time steps is stored in the cell state. The layer updates the cell state at each time step by adding or removing the information. Finally, gates are used by the layer to control the updates.

The parameters of an LSTM layer are learnable and given as the bias b (Bias), the recurrent weights R (RecurrentWeights), and the input weights w (InputWeights). The matrices b, w and R are concatenations of the bias, the recurrent weights, and the input weights of each component, respectively. The following equations illustrate how these matrices are concatenated:

(24) $$\begin{array}{c} b=\left[\begin{array}{c} b_{i}\\ b_{f}\\ b_{g}\\ b_{o} \end{array}\right],R=\left[\begin{array}{c} R_{i}\\ R_{f}\\ R_{g}\\ R_{o} \end{array}\right],w=\left[\begin{array}{c} w_{i}\\ w_{f}\\ w_{g}\\ w_{o} \end{array}\right], \end{array}$$

where *o*, *g*, *f* and *i* denote the output gate, cell candidate, forget gate and input gate, respectively. At time step *t*, the cell state is updated as:

(25) $$\begin{array}{c} c_{t}=f_{t}⊙c_{t-1}+i_{t}⊙g_{t}, \end{array}$$

where the symbol ⊙ represents the element-wise multiplication of vectors, i.e., the Hadamard product. At time step *t*, the hidden state is updated as:

(26) $$\begin{array}{c} h_{t}=o_{t}⊙\sigma_{c}\left(c_{t}\right), \end{array}$$

where $\sigma_{c}$ represents the state activation function. The LSTM layer function uses the softsign function in order to compute the state activation function, i.e.,

(27) $$\begin{array}{c} \mathrm{softsign}\left(x\right)=\frac{x}{1+|x|}. \end{array}$$

The following formulas describe the components at time step *t*. The hidden and cell states of the layer are controlled by the following components:

- Output gate (*o*): controls the cell state level that is added to the hidden state;
- Cell candidate (*g*): adds information to the cell state;
- Forget gate (*f*): controls the level of cell state reset (forget);
- Input gate (*i*): controls the level of cell state update.
  (28) $$\begin{array}{cc} \;\mathrm{Output}\;\mathrm{gate}\;:\;\; & o_{t}=\sigma_{g}\left(w_{o}x_{t}+R_{o}h_{t-1}+b_{o}\right) \end{array}$$
  (29) $$\begin{array}{cc} \;\mathrm{Cell}\;\mathrm{candidate}\;:\;\; & g_{t}=\sigma_{c}\left(w_{s}x_{t}+R_{g}h_{t-1}+b_{g}\right) \end{array}$$
  (30) $$\begin{array}{cc} \;\mathrm{Forget}\;\mathrm{gate}\;:\;\; & f_{t}=\sigma_{g}\left(w_{f}x_{t}+R_{f}h_{t-1}+b_{f}\right) \end{array}$$
  (31) $$\begin{array}{cc} \mathrm{Input}\;\mathrm{gate}:\;\; & i_{t}=\sigma_{g}\left(w_{i}x_{t}+R_{i}h_{t-1}+b_{i}\right) \end{array}$$

The gate activation function is denoted by $\sigma_{g}$ in these calculations. The hard-sigmoid function used by the LSTM layer function is given by:

$$\sigma_{x}=\left\{\begin{array}{cc} 1 & \;\mathrm{if}\;x>2.5\\ 0 & \;\mathrm{if}\;x<-2.5\\ 0.2x+0.5 & \;\mathrm{if}\;-2.5\leq x\leq 2.5 \end{array}\right.$$

## 5. Results and Discussion

### 5.1. Synthetic Data Results

**Figure 4:**
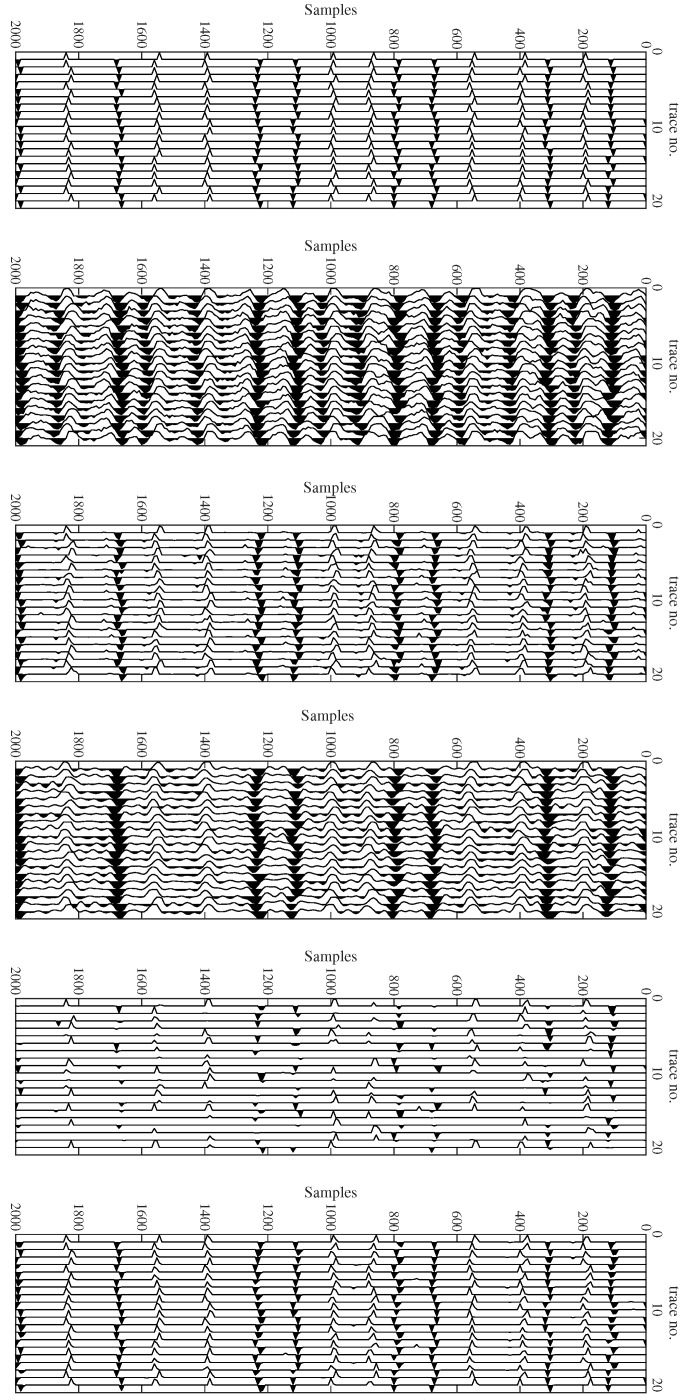
Deconvolution result for Q = 0.8 Y. Top to bottom: True reflectivity, received data with SNR = 10 dB, BP method, SD method, proposed method (initial), proposed method (refined).

In the synthetic simulation, we generate 20 traces using the reflectivity shown in Figure 4, where the sampling frequency is 500 Hz. The original traces shown in Figure 4 are the result of the convolution between the Ricker wavelet, having a center frequency of 40 Hz and a phase shift of 50 degrees, and the reflectivity series. The noise is modeled as the additive white Gaussian noise (AWGN) with an SNR of 10 dB, whereas the SNR is defined for the additive model y=x+z as:

(32) $$\mathrm{SNR}=10\log_{10}\left(\frac{{∥x∥}_{2}^{2}}{{∥z∥}_{2}^{2}}\right),$$

where x and z are the signal part and noise part, respectively.

For the SD method, a deconvolution filter of length 51 is used. The filter is initialized with a solitary spike that is located approximately in the middle of the filter used for deconvolution. Other parameters used in the experiments are given as follows: α=0.2, $\epsilon_{f}=$ 1, step size μ= 0.02, the stopping criterion is marked as 1000 iterations, the hybrid norm parameter $\epsilon_{k}$ is set to 0.005, and the regularization parameter λ is fixed to 2. The SD method’s iteration count is set to 800 to ensure that the algorithm reaches a steady state. A number of efficient basis pursuit solvers are available to solve the embedded basis pursuit problem in the BP method. The SPGL1 package developed by [19] is utilized here to solve this $\ell_{1}$ minimization problem.

The estimated reflectivity series using different algorithms are depicted in Figure 4. We can see that the proposed method gives the spikiest result with the least noise, which is the most desirable for interpretation.

**Figure 5:**
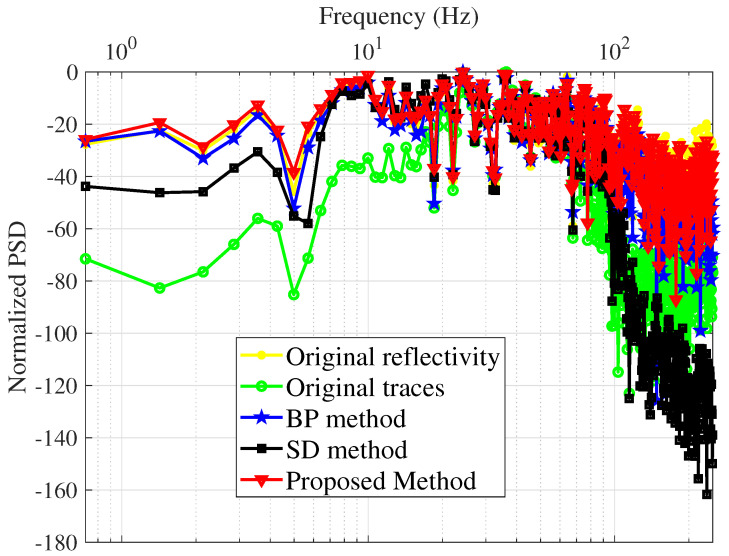
Normalized PSD of the original synthetic traces, original reflectivity, and estimated reflectivity.

Power spectrum density (PSD) is used to evaluate the quality of the proposed method as well, presented in Figure 5. Among the three schemes, the proposed technique provides the flattest PSD, which is pretty close to the true PSD of the reflectivity series.

**Figure 6:**
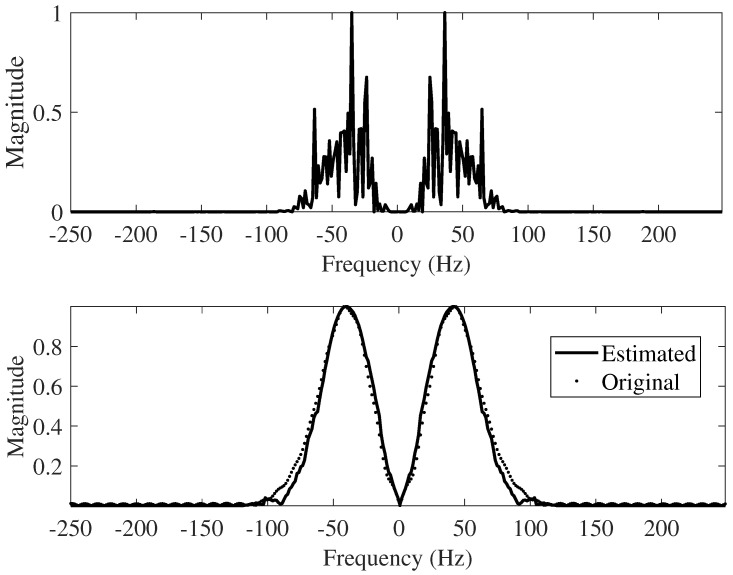
Frequency-domain views of the wavelet recovery process (Q = 0.8 Y). Top: frequency-domain first iteration; Bottom: frequency-domain final iteration.

**Figure 7:**
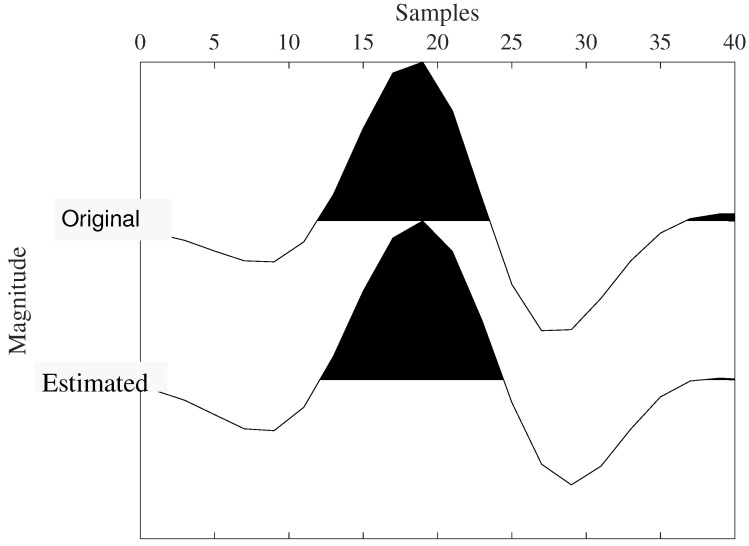
Recovered and true wavelets.

The first iteration and the last iteration of the proposed method are shown in Figure 4. The intermediate iteration, with the recovery of the wavelet, is detailed in Figure 6 and Figure 7. Note the exact match after the final iteration in the frequency domain. Upon obtaining the time-domain wavelet, a chunk of it (consisting of 51 samples and designated as a window) is used to refine the reflectivity in the BD algorithm’s final iteration.

To compare the performance of the proposed algorithm with the SD method and the BD method, two figures of merit are used: relative reconstruction error (RRE) and signal-to- reconstruction error ratio (SRER). They are defined by:

(33) $$\begin{array}{c} \mathrm{RRE}=\frac{{\left(r-\hat{r}\right)}^{T}\left(r-\hat{r}\right)}{{\hat{r}}^{T}r} \end{array}$$

and

(34) $$\begin{array}{c} \mathrm{SRES}=10\log_{10}\frac{{∥r∥}_{2}^{2}}{∥r-\hat{r}{∥}_{2}^{2}}. \end{array}$$

**Figure 8:**
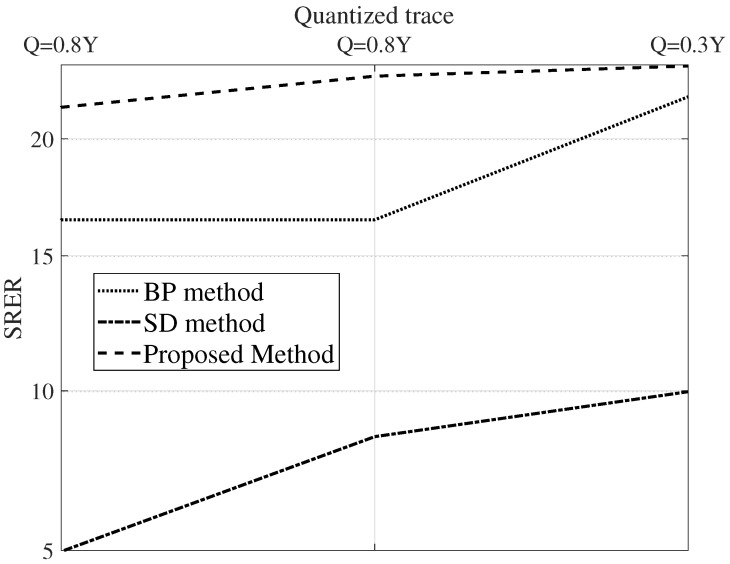
Normalized correlation coefficient (γ) versus SNR with 20 traces.

**Figure 9:**
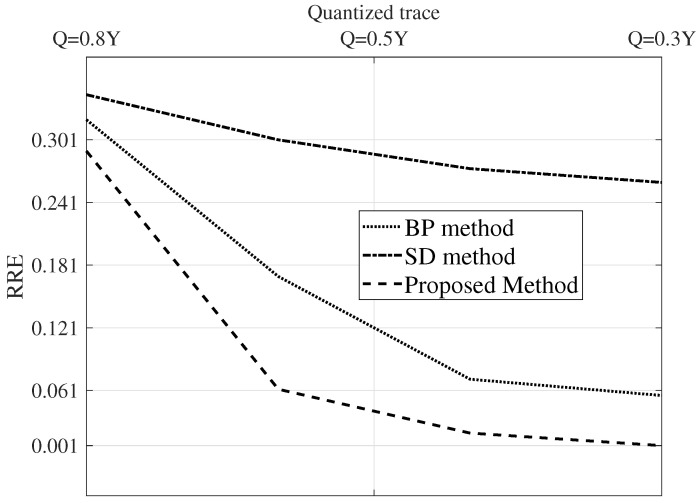
Quality (*Q*) versus SNR with 20 traces.

Figure 8 and Figure 9 show the parameters SRES and RRE for various SNR levels. As can be seen from these figures, the proposed algorithm outperforms the SD method and BD method algorithms in terms of SRES and RRE for all noise levels.

**Figure 10:**
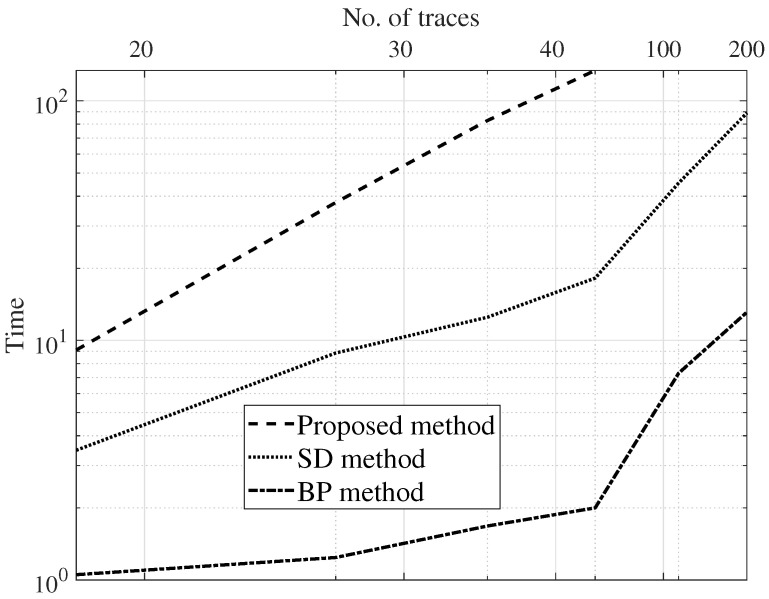
Simulation time versus number of traces.

For complexity analysis, the simulation time taken by the estimation methods is charted against the count of the traces. Figure 8, Figure 9 and Figure 10 are the results of a Monte Carlo simulation performed with 20 different realizations of noise and seismic reflectivity. The BP method takes much less time, as depicted in Figure 10. We can see that when we simulate 200 traces, the BP method’s computation takes approximately 12% of the time needed for the SD method. In addition, for a simulation with this many traces, the SD method requires a prohibitively large amount of memory, so it fails to run on the same laptop machine. The computation time of the proposed approach is a bit high due to the training phase. However, in the proposed method, the number of parameters to tweak is very limited.

### 5.2. Field Data Results

The results of the SD, BD, and proposed methods are applied on a field seismic data set, and the results are presented in this section. The field seismic data set was obtained from the Line ID 51-101, Legacy Data Archive by USGS (1976), National Petroleum Reserve, Alaska (NPRA).

For the field data set, we run the SD method in windows with a duration of 0.35 s in time with the total number of traces set as 150. For the SD method, the deconvolution filter size is fixed to 21 samples, and a spike is marked at the filter’s center for the purpose of initialization. Furthermore, the step size for the SD method algorithm is set to μ=0.1. For the BP method, the window is the same as in the SD method. The deconvolution filter used for the SD method is estimated for the entire field data set. All three techniques’ remaining parameters are identical to those used in the synthetic data test.

**Figure 11:**
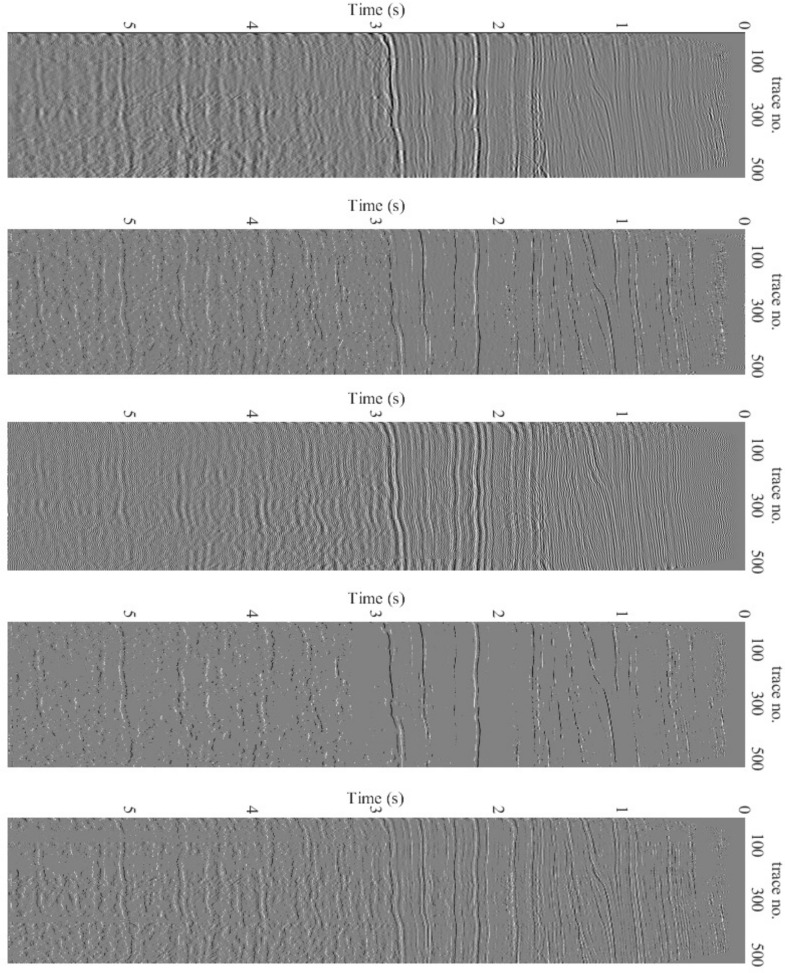
Deconvolution results for (Q = 0.8 Y). Top to bottom: Received data for 6 s and 500 traces, BP method, SP method, Proposed (first iteration) and Proposed (last iteration).

**Figure 12:**
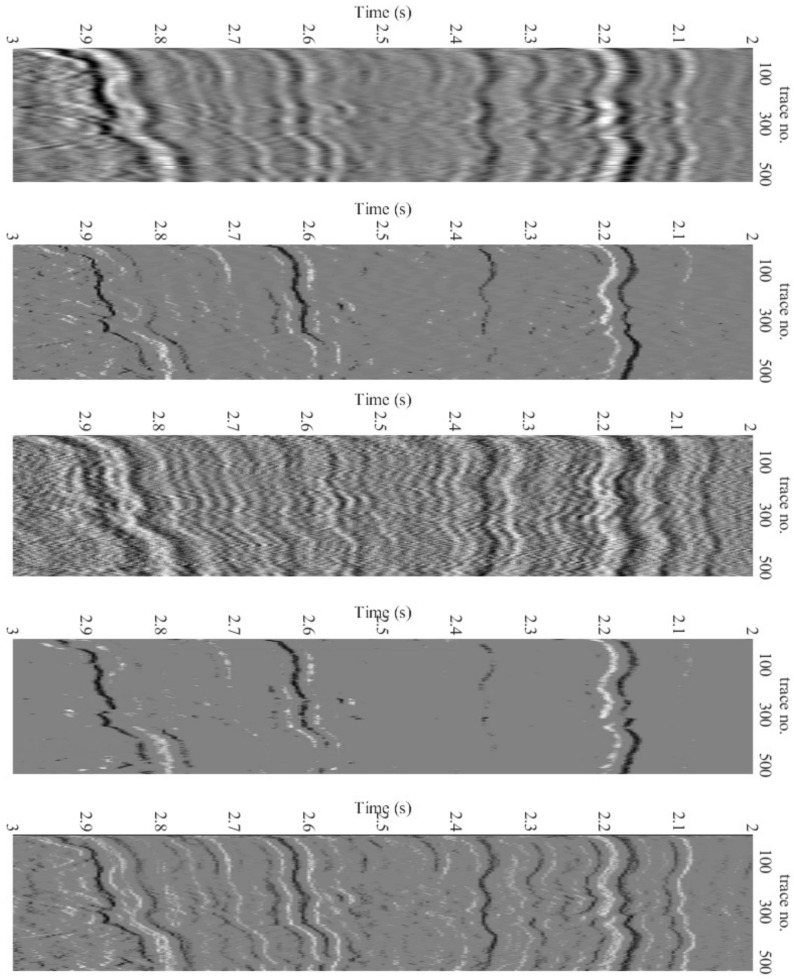
Deconvolution results. Top to bottom: Subset of received data for 2≤t≤3 s and 500 traces, BP method, SP method, Proposed (first iteration) and Proposed (last iteration).

**Figure 13:**
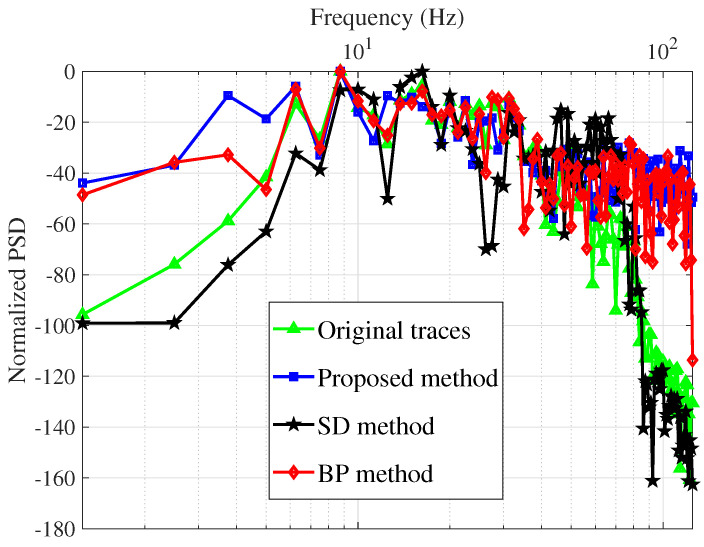
Normalized PSD of the original field traces, original reflectivity, and estimated reflectivity.

Figure 11 shows the deconvolution results for the three algorithms together with the recorded data. Figure 12 depicts the relevant information of a zoomed-in field data section prior to and after the processing, i.e., deconvolution. The proposed technique has a more spiky deconvolution outcome than the other two algorithms, as shown by these results. The respective normalized power spectral densities (PSD) are shown in Figure 13. In terms of the power densities, the SD method provides significant gain in the 40–70 Hz band, whereas the BD method has a relatively flat normalized PSD. Figure 11 and Figure 13 are plotted after applying RMS automatic gain control with a window length of 0.1 s to compensate for the lost amplitude after deconvolution.

**Figure 14:**
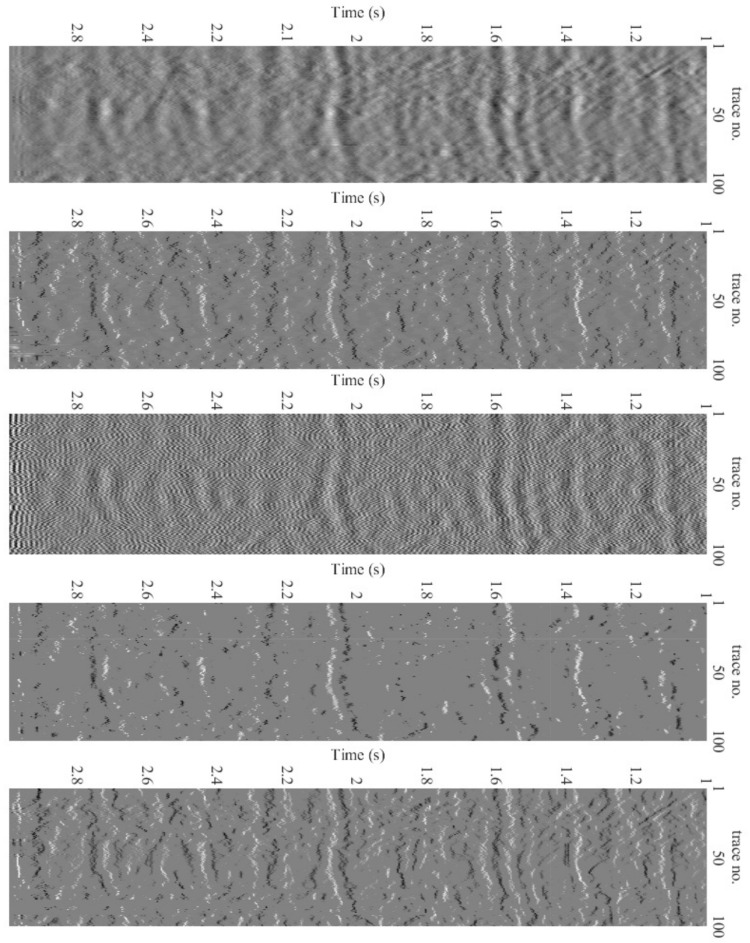
Deconvolution results. Top to bottom: 100 traces of raw field data set, BP method, SP method, Proposed (first iteration), and Proposed (last iteration).

Finally, as shown in Figure 14, another field data set consisting of a two-dimensional (2D) land line from east Texas, USA [20], is used for a performance comparison. The graph demonstrates the superior performance of the proposed method in comparison to the other methods.

In the BP method, several parameters require attention. First, τ in Equation (19) is the parameter that controls the number of peaks (i.e., sparsity) in the initial estimate of the per channel reflectivity. If we pick τ=1, the solution from (19) is a trivial solution that pinpoints only one peak at the highest value of $x_{j}\left[n\right]$. On the other hand, if τ is too large, the per channel estimate of the reflectivity is not sparse. Empirically, a value of 1.6 works well over a wide range of SNR for both synthetic and field data.

## 6. Conclusions

This study addresses the issue of recovering sparse reflectivity sequences from randomly quantized seismic data and thus proposes a robust reconstruction approach based on a neural network. The technique is beneficial for compressing massive amounts of seismic data. In experiments with both synthetic and field data sets, the proposed scheme significantly improves the solution’s robustness to changes in quantization error and thus enhances the visual saliency of properly salvaged reflectivity impulses compared with existing methods. During the training phase, only a small number of fundamental signals are chosen in order to reduce the time. In the experiments with the synthetic data, since the true reflectivity is known, the quality of the recovery is easy to evaluate. The proposed method is effective and robust against the quantization noise, i.e., for a wide range of SNRs, it outperforms the BD and SD methods in terms of relative reconstruction error and signal-to-reconstruction error ratio. All this is achieved at the expense of the computation time, which is a bit high due to the training phase. Finally, in the proposed method, the number of parameters to tweak is very limited, making the scheme simple to implement in real-world applications.

## Data Availability

The data are available by contacting the corresponding author.
